# Is poor chewing ability a risk factor for malnutrition? A six-year longitudinal study of older adults in Sweden

**DOI:** 10.1016/j.jnha.2025.100554

**Published:** 2025-04-02

**Authors:** Duangjai Lexomboon, Abhishek Kumar, Sara Freyland, Weili Xu, Gunilla Sandborgh-Englund

**Affiliations:** a Academic Center for Geriatric Dentistry, Stockholm, Sweden; b Department of Dental Medicine, Karolinska Institutet, Huddinge, Sweden; c Institute of Environmental Medicine, Division of Biostatistics, Karolinska Institutet, Stockholm, Sweden; d Aging Research Center, Department of Neurobiology, Care Sciences and Society, Karolinska Institutet, Stockholm, Sweden

**Keywords:** Weight loss, Nutritional status, Oral health, Masticatory, Longitudinal study, Living arrangement

## Abstract

**Objective:**

To investigate if poor chewing ability increases the risk of malnutrition and to compare its impact with other contributing factors.

**Design:**

Longitudinal observational study.

**Setting:**

Population-based survey.

**Participants:**

1,596 community dwelling individuals aged 60 years or older who participated in the Swedish National Study on Aging and Care at Kungsholmen in 2001–2004 (baseline) and in 2007–2011 (follow-up) and were not at risk for malnutrition nor malnourished at baseline.

**Measurements:**

The exposures were baseline chewing ability and change in chewing ability at follow-up. The primary outcome was malnutrition risk or being malnourished, as assessed by the Mini Nutritional Assessment Short-Form. The secondary outcome was weight loss over 10% at follow-up. Logistic regressions assessed the associations between the exposures and the outcomes. The average marginal effects (percentage points) compared the effect of the exposure versus covariates on outcome probability.

**Results:**

150 (9.4%) reported having difficulty chewing hard food, while 191 (12.0%) had persistent difficulties chewing hard food or lost the ability during the follow-up. At the time of follow-up, 212 (13.3%) were at risk or malnourished, while 179 (11.2%) had weight loss of more than 10%. Self-reported difficulty chewing hard food increased the odds of being at risk or malnourished at follow-up (OR = 1.64, 95% CI = 1.06, 2.53) and having weight loss of more than 10% (OR = 1.72, 95% CI = 1.10, 2.68). Individuals who had persistent difficulty chewing hard food or lost the ability to chew hard food during the follow-up period were more likely to be at risk or malnourished (OR = 1.87, 95% CI = 1.26, 2.79) or had a weight loss of more than 10% (OR = 1.73, 95% CI = 1.12, 2.65). Having difficulty chewing hard food at baseline increased the probability of the two outcomes by approximately 6 percentage points, whereas the covariates increased or decreased the probabilities by 4–16 percentage points.

**Conclusion:**

Poor chewing ability may be a low-risk factor for malnutrition in older individuals. Self-reported difficulty chewing hard food during dental visits should be addressed.

## 1 Introduction

Malnutrition, characterized by deficiencies in energy and nutrient intake, is common among older adults, with prevalence rates ranging from 5% to 50% depending on the population and setting [1]. This condition poses significant health risks, including increased susceptibility to infections, frailty, reduced quality of life, and even higher mortality rates [[2], [3], [4]]. Studies have shown that malnutrition can lead to early death in older individuals, particularly in those with underlying health conditions or functional impairments [3,5,6]. As malnutrition becomes more prevalent with age, it is essential to explore all potential contributing factors, including oral health, to better understand and mitigate its impact on the older population.

Studies have shown that nutritional status is influenced by multiple independent factors (determinants), including lifestyle habits, loneliness, social isolation, marital status, educational level, socioeconomic status, financial hardships, and place of residence [5,7]. Besides these independent factors, chewing problems caused by oral health issues such as missing teeth, poorly fitting dentures, or reduced masticatory efficiency are another significant contributor to malnutrition [[8], [9], [10]]. However, the evidence on association between chewing function and malnutrition remains unclear [11]. Our recent systematic review examined the influence of objectively assessed chewing function on swallowing, physiologic and pathologic processes in the gastrointestinal tract, and nutrition-related factors. Specifically, it was inferred that impaired chewing function is a determinant of nutritional status [12]. There was, however, a moderate to very low certainty of evidence to suggest that chewing function contributes to nutritional status. All except three of the studies included in the review on the effect of chewing ability were cross sectional and intervention studies. Therefore, the study samples were limited; one study included men only, and the other two involved specific groups of patients receiving prosthodontic treatment [[13], [14], [15]].

On the contrary, a systematic review focused on self-reported chewing ability found that poor chewing ability was not a determinant of malnutrition in older adults [7]. The review was limited to prospective cohort studies, with all except one study having a one or two-year follow-up period. In the absence of acute major illness or trauma, malnutrition in older individuals, particularly in community settings, is a chronic condition. The metabolism can adapt to compensate for food deprivation, nutrient malabsorption, and high energy expenditure caused by inflammation [1]. The short follow-up time might have contributed to the non-significant findings. However, there is a lack of long-term observational studies to determine whether or not poor chewing ability contributes to a risk of malnutrition.

We hypothesize that while poor chewing ability may play a role in the development of malnutrition, its contribution is likely limited, and the effects may take a long time to manifest. This six-year longitudinal study aims to investigate whether chewing ability increases the risk of malnutrition, and to compare its impact with other contributing factors. Additionally, as chewing ability may change over time, the study will assess malnutrition risk in individuals experiencing different levels of change in their chewing ability.

## 2 Materials and methods

### 2.1 Study design and sample

**Fig. 1:**
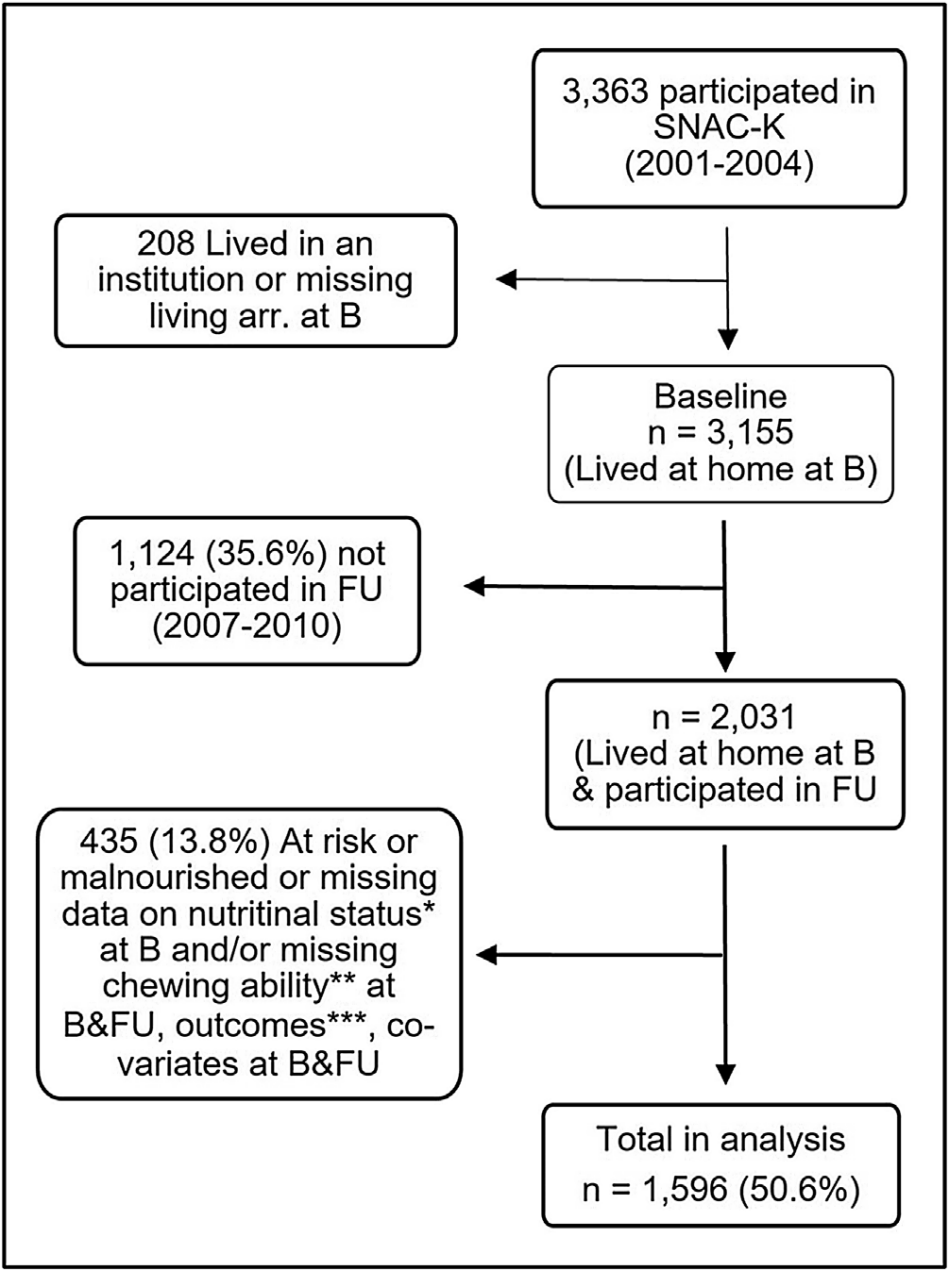
Flow diagram. SNAC-K = Swedish National study on Aging and Care at Kungsholmen; B = Baseline; FU = Follow-up; living arr. = Living arrangement. * Assessed by MNA-SF at baseline; ** Ability to chew hard food; *** Outcomes are At risk or malnourished (assessed by Mini Nutritional Assessment–Short Form [MNA-SF]) and weight loss of >10% at follow-up; Co-variates = Socio-demographics, living arrangement, and diseases that can affect nutritional status.

This study is a six-year longitudinal cohort study, using data from the Swedish National study on Aging and Care at Kungsholmen (SNAC-K). The SNAC-K is a population-based, multidisciplinary longitudinal survey on aging and health of individuals aged 60+ who lived in Kungsholmen, a part of Stockholm County, Sweden [16]. The SNAC-K participants were selected by random sampling, stratified by age and year of the surveys. The inclusion criteria in this study were individuals who lived at home and participated in the first SNAC-K cohort during 2001–2004 (baseline year). Of the total 3,363 randomly selected individuals, 3,155 lived at home and were eligible to participate in the SNAC-K data collections [17]. Those who did not participate in the six-year follow-up survey in 2007–2010, and those who were being at risk or malnourished at baseline as assessed by Mini Nutritional Assessment Short Form (MNA-SF), and/or those who had missing data on chewing ability at baseline and/or follow-up, outcome variables, and/or covariates at baseline and follow-up were excluded (Fig. 1). The outcomes were nutritional status at the six-year follow-up, as assessed by MNA-SF, and percentage weight loss. Covariates included socio-demographics, living arrangements, and diseases that can have an effect on nutritional status. A total of 1,596 (50.6%) were included in the analysis.

### 2.2 Ethical aspects

The SNAC-K was approved by the Ethics Committee at Karolinska Institute and the Regional Ethical Review Board in Stockholm (01-114, 2001-06-18, 04-929/3, 2005-01-19, 2010/44, 2010-06-09, 2013/828-31/3). The study is reported according to the Strengthening the Reporting of Observational Studies in Epidemiology (STROBE) statement guidelines.

### 2.3 Data collection

The pseudonymized data were retrieved from the SNAC-K database. During the baseline and follow-up surveys, SNAC-K data collection consisted of medical examination, blood sampling, cognitive assessments, and an interview at the SNAC-K center conducted by teams of a physician, a registered nurse, and a psychologist. Data on diseases were retrieved from the National Patient Registry [16].

### 2.4 Chewing ability assessment

Chewing ability was assessed based on self-reported ability to chew hard food. A registered nurse asked the question and clarified the answers options. Participants were asked, “Can you chew hard food such as hard bread or apples?” with three possible response options: (1) “Yes, without difficulty”, indicating no issues with chewing hard food; (2) “Yes, but I must be careful”, reflecting some difficulty or the need to adjust chewing habits, such as taking smaller bites or chewing more slowly to avoid discomfort; and (3) “No, not at all”, indicating a complete inability to chew hard food [18].

### 2.5 Assessment of the outcomes

The primary outcome is nutritional status as assessed by MNA-SF, while the secondary outcome is percentage weight change from baseline to the six-year follow-up.

MNA-SF is a validated tool for identifying older adults at risk of or being malnourished, and it has been used in all living settings and in various populations [19]. MNA-SF consists of six domains: (1) Decline in food intake during the last three months (score 0–2); (2) Weight loss during the last three months (score 0–3); (3) Mobility (score 0–2); (4) Psychological stress or acute diseases in the last three months (score 0–2); (5) Psychological problems, that is, dementia and/or depression (score 0–2); and (6) Body Mass Index (score 0–3). The total possible score ranges from 0 to 14. The score for each domain in this study was assigned according to a previous study using SNAC-K data [20]. For domain 1 Decline in food intake during the last 3 months, a score of 2 was assigned to the answer with normal or increased appetite, a score of 1 to the answer with poor appetite, and a score of 0 was assigned to the answer with very poor or no appetite. The scores for domain 2 Weight loss during the last 3 months and domain 4 Psychological stress or acute diseases in the last 3 months were assigned based on its answer, which is the same as in the original MNA-SF. For domain 3 Mobility, scores were assigned based on three questions: ability to move in and out of bed or chair unassisted, ability to move inside the house independently, and ability to move outside the house independently. Domain 5 Neuropsychological problems (that is dementia and depression) were assessed by medical examinations and by medical records, and depression severity was determined using the the International Classification of Diseases, 10th Revision (ICD-10), which was obtained from the National Patient Registry. Domain 6 Body Mass Index was calculated by dividing weight in kilograms by squared height in meters, and scores were assigned based on this value. Weight and height were measured using a standard scale while people wore light clothing and no shoes. When weight and height were not measured on a scale, self-reported values were used. The total score was categorized into normal (12–14 points), at risk of malnutrition (8–11 points), and malnourished (0–7 points).

Weight loss of more than 10% of the baseline weight over six years was used as an indicator of malnutrition [2]. Weight loss of more than 10% was calculated as the weight difference between the six-year follow-up and the baseline divided by the weight at the baseline.

### 2.6 Covariates and nutritional status at baseline

Socio-demographics include age, sex, and education. However, income data was not available. Age was not used as a continuous variable because the SNAC-K population was drawn at random from individuals aged 60, 66, 72, 78, 81, 84, 87, 90, 96, and 99+ years. Age was categorized into 60−69, 70−79, and 80+ years old. Education was categorized into elementary school, high school, and university.

Loneliness and living situation are reported to be associated with nutritional status of older adults [21]. Living arrangement was constructed from two questions: type of housing (group housing or private housing) and living alone or with another person. Since the study population did not include individuals living in an institution at baseline, living arrangement was categorized into living at home alone and living at home with someone.

Data on diseases that could impact nutritional status were obtained from the National Patient Registry and were identified by the ICD-10. The relevant conditions include dysphagia (code R13), malabsorption, diseases of the digestive system (codes K90- K93), and cancer (codes C00-C96) [7]. Xerostomia was assessed by self-reported symptoms of dry mouth and was categorized dichotomously as “Yes” (presence of symptoms) or “No” (absence of symptoms).

Individuals' nutritional status was assessed using their baseline BMI.

### 2.7 Data analyses

The change in chewing ability was categorized into “Consistently good”, “Improved”, “Deteriorated”, and “Persistent problem”. The change in living arrangement was categorized into “Living alone”, “Co-living”, “Transition to co-living”, “Transition to living alone”, “Living alone to institution”, and “Co-living to institution”. The definitions of these categories are shown in Supplementary Table S1. Baseline characteristics are presented as counts and percentages for the categorical variables of interest. Summaries were provided for the entire study population and stratified by chewing ability at baseline. The associations were assessed using Chi-square tests. The mean and standard deviation of BMI were calculated, and the difference between groups was assessed using one-way ANOVA.

Logistic regression was used for the analyses, with MNA-SF and weight loss greater than 10% as the outcomes and chewing ability at baseline and change in chewing ability as the exposures. Crude and adjusted models were run for each outcome and exposure. Since there were no individuals who could not chew hard food at all and who were also malnourished at the follow-up, the MNA-SF at baseline and the follow-up were dichotomized into “Normal” and “At risk or malnourished”. The chewing ability was dichotomized into “No problem” and “Must be careful or cannot chew hard food at all”. For the change in chewing ability, “Deteriorated” and “Persistent problem” were combined due to low frequencies. Only covariates at baseline that were significantly associated with the exposure (chewing ability at baseline) and the main outcome (MNA-SF) were included in the adjusted models (Supplementary Table S2). Therefore, the models with chewing ability at baseline as the exposure were adjusted for baseline variables age, sex, education, and living arrangement, or change in living arrangement for the models with change in chewing ability as the exposure. To demonstrate the size of the effect from an exposure (or a covariate) on the probability of the outcomes, the average marginal effects for the adjusted models were calculated by estimating the difference between the predicted outcome when an exposure (or covariate) was set to a given level (category) and the predicted outcome when the exposure (or covariate) was set to its reference level (category) for all subjects and then averaging. The Average Marginal Effects show the difference in the probability of the outcome (at risk or malnourished or having >10% weight loss at follow-up) between a category in the exposure (or a covariate) and its reference category, such as a woman versus man (reference category). The probability differences were expressed in percentage points (pp). All analyses were conducted using STATA (StataCorp. 2023. *Stata Statistical Software: Release 18*. College Station, TX: StataCorp LLC) and *p*-values below 0.05 were considered statistically significant.

## 3 Results

### 3.1 Baseline characteristics

Baseline characteristics of the included individuals by chewing ability at baseline are shown in Table 1. A higher proportion of the individuals who reported “Must be careful” or “Cannot chew hard food at all” were older, had a lower level of education, lived alone, had dysphagia, and had xerostomia, compared to the individuals who reported no problem chewing hard food. There were no statistically significant differences in BMI. The prevalence of malabsorption and/or gastrointestinal diseases was low, with no statistically significant difference between individuals with different chewing ability. Only 1 (0.1%) had mild to moderate dementia, and 1 (0.1%) had severe depression (data not shown in Table 1). The two participants had no trouble chewing hard foods. All 1,596 individuals reported that they could get in and out of bed or chairs and could move inside and outside the house independently.

**Table 1:** Baseline characteristics of the study population and the outcome prevalence by the ability to chew hard food.

		Ability to chew hard food, n (%)				
		Total	No problem	Must be careful	Cannot at all	p-value
Total n (%) *		1,596	1,446 (90.6%)	88 (5.5%)	62 (3.9%)	
Age (year)	60−69	893 (56.0%)	837 (57.9%)	37 (42.0%)	19 (30.6%)	<0.001
	70−79	492 (30.8%)	433 (29.9%)	30 (34.1%)	29 (46.8%)	
	80+	211 (13.2%)	176 (12.2%)	21 (23.9%)	14 (22.6%)	
Sex	Men	615 (38.5%)	568 (39.3%)	25 (28.4%)	22 (35.5%)	0.111
	Women	981 (61.5%)	878 (60.7%)	63 (71.6%)	40 (64.5%)	
Education	Elementary school	310 (19.4%)	264 (18.3%)	26 (29.5%)	20 (32.3%)	<0.001
	High school	683 (42.8%)	606 (41.9%)	49 (55.7%)	28 (45.2%)	
	University	603 (37.8%)	576 (39.8%)	13 (14.8%)	14 (22.6%)	
Living arrangement	Alone	736 (46.1%)	646 (44.7%)	53 (60.2%)	37 (59.7%)	0.002
	With someone	860 (53.9%)	800 (55.3%)	35 (39.8%)	25 (40.3%)	
Xerostomia	No	1578 (98.9%)	1431 (99%)	88 (100%)	59 (95.2%)	0.012
	Yes	18 (1.1%)	15 (1.0%)	0 (0%)	3 (4.8%)	
Dysphagia	No	1595 (99.9%)	1446 (100%)	88 (100%)	61 (98.4%)	<0.001
	Yes	1 (0.1%)	0 (0%)	0 (0%)	1 (1.6%)	
Malabsorption & GI diseases	No	1588 (99.5%)	1438 (99.4%)	88 (100%)	62 (100%)	0.659
	Yes	8 (0.5%)	8 (0.6%)	0 (0%)	0 (0%)	
Cancers	No	1513 (94.8%)	1374 (95%)	82 (93.2%)	57 (91.9%)	0.440
	Yes	83 (5.2%)	72 (5%)	6 (6.8%)	5 (8.1%)	
BMI	Mean ± SD	26.3 ± 3.7	26.3 ± 3.7	26.8 ± 3.8	26.8 ± 3.8	0.216
MNA-SF at follow-up	Normal	1384 (86.7%)	1268 (87.7%)	65 (73.9%)	51 (82.3%)	<0.001
	At risk	198 (12.4%)	167 (11.5%)	20 (22.7%)	11 (17.7%)	
	Malnourished	14 (0.9%)	11 (0.8%)	3 (3.4%)	0 (0%)	
Weight loss at follow-up	No loss or Loss ≤10%	1417 (88.8%)	1298 (89.8%)	68 (77.3%)	51 (82.3%)	<0.001
	Loss >10%	179 (11.2%)	148 (10.2%)	20 (22.7%)	11 (17.7%)	

MNA-SF = Mini Nutritional Assessment-Short Form; BMI = Body Mass Index; GI diseases = Gastrointestinal diseases; Outcomes = MNA-SF at follow-up and Weight loss at follow-up.

* Percent row.

### 3.2 Chewing ability and malnutrition

At the end of the six-year follow-up, the incidence of being at risk for malnutrition and being malnourished were 12.4% and 0.9%, respectively, as assessed by MNA-SF, and 11.2% had weight loss of more than 10% (Table 1).

Crude and adjusted logistic models of the association between ability to chew hard food and nutritional status are shown in Table 2 and Supplementary Tables S5 and S6. Difficulty chewing hard food was associated with an increased risk of being at risk or malnourished at follow-up, as measured by MNA-SF (OR = 1.64, 95% CI = 1.06, 2.53), and with a weight loss of more than 10% (OR = 1.72, 95% CI = 1.10, 2.68).

**Table 2:** Odds ratios of logistic regression models for the association of chewing ability at baseline and change in chewing ability (as the exposures) with being at risk or malnourished (assessed by MNA-SF) and percentage weight loss (as the outcomes) at 6-year follow-up.

		At risk or malnourished (MNA-SF)				Weight loss >10%			
	n (%)	Crude		Adjusted*		Crude		Adjusted*	
	Total = 1,596	OR	95% CI	OR	95% CI	OR	95%CI	OR	95%CI
Ability to chew hard food at baseline									
No problem	1,446 (90.6%)	1.00		1.00		1.00		1.00	
Must be careful or Cannot	150 (9.4%)	**2.09**	**(1.38, 3.16)**	**1.64**	**(1.06, 2.53)**	**2.28**	**(1.49, 3.51)**	**1.72**	**(1.10, 2.68)**
Change in ability to chew hard food									
Consistently good**	1,319 (82.6%)	1.00		1.00		1.00		1.00	
Improved	86 (5.4%)	**1.95**	**(1.12, 3.40)**	1.71	(0.97, 3.10)	1.36	(0.70, 2.63)	1.15	(0.59, 2.24)
Declined or Persistent problem**	191 (12.0%)	**2.58**	**(1.78, 3.74)**	**1.87**	**(1.26, 2.79)**	**2.49**	**(1.68, 3.70)**	**1.73**	**(1.12, 2.65)**

MNA-SF = Mini Nutritional Assessment-Short Form; OR = Odds Ratio; CI = Confidence Interval.

* Adjusted for age, sex, education, living arrangement (chewing ability as the exposure) or change in living arrangement (change in chewing ability as the exposure).

** Four cases who moved from co-living to an institution, two with consistently good chewing ability and two with declined or persistent poor chewing ability, were excluded from the regressions that had weight loss >10% outcome because none had weight loss of >10% (no cases to compare to the reference weight loss ≤10%).

At follow-up, 1,319 (82.6%) had consistently good chewing ability, 86 (5.4%) had improved chewing ability, and 191 (12.0%) had persistently poor or deteriorated chewing ability (Supplementary Table S4). The majority continued to live at home either alone (658, 41.2%), or with someone (746, 46.7%). While 110 (6.9%) changed from living at home with someone to living alone, a smaller proportion (62, 3.9%) changed from living alone to living with someone at home. A small proportion (16, 1.0%) transitioned from living at home alone to living in an institution, and only 4 (0.3%) transitioned from living at home with someone to living in an institution. When comparing the changes in chewing ability over time, the odds ratio for being at risk or malnourished (as measured by MNA-SF) was significantly higher (OR = 1.87, 95% CI = 1.26, 2.79) in individuals who had persistently poor or deteriorated chewing ability, compared to those who had consistently good chewing ability (Table 2, Supplementary Tables S7 and S8). Similarly, the odds ratio for having weight loss of more than 10% was significantly higher (OR = 1.73, 95% CI = 1.12, 2.65) in the individuals with persistently poor or deteriorated chewing ability.

### 3.3 Comparing the effects of chewing ability and covariates on malnutrition

**Fig. 2:**
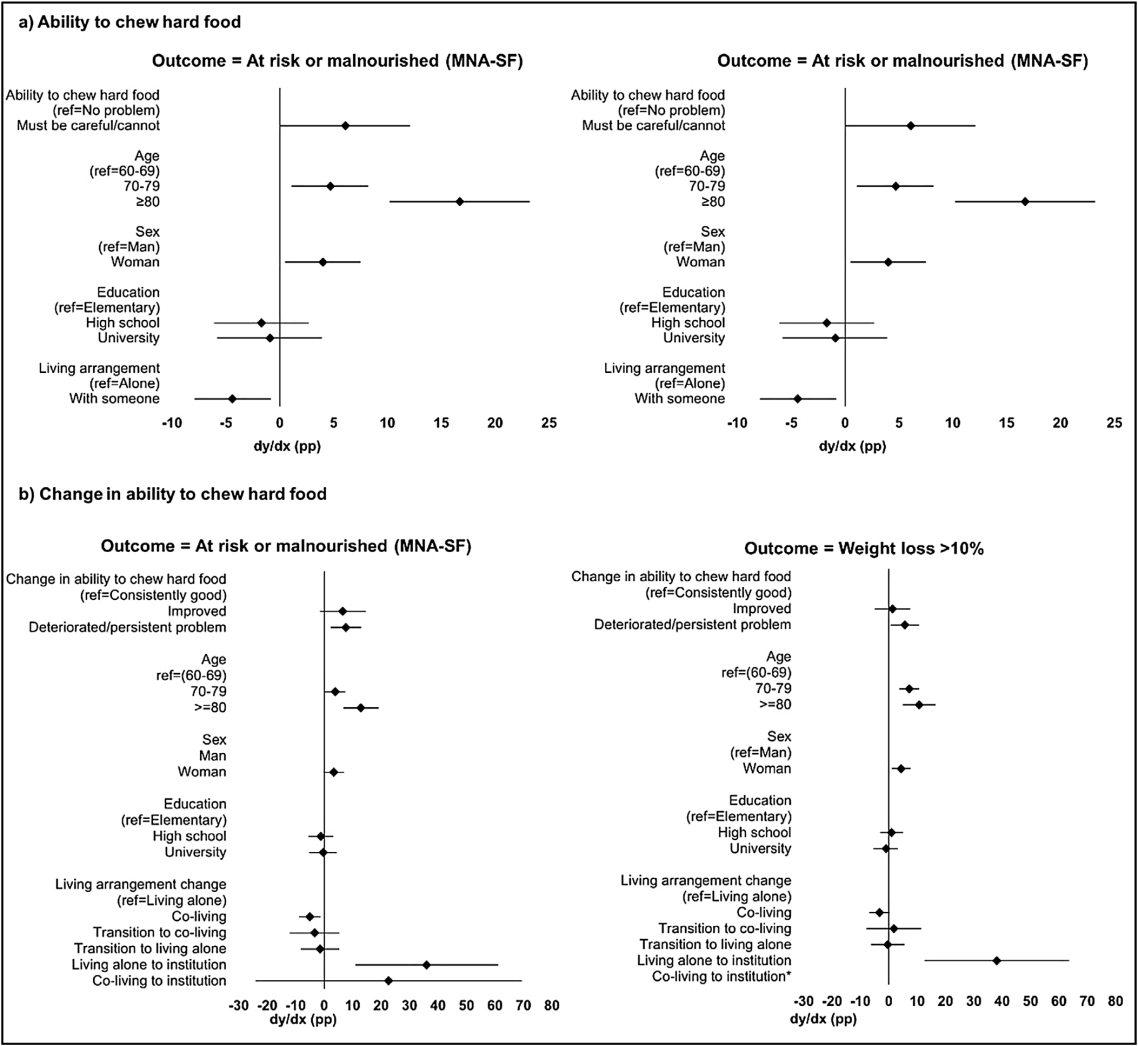
Average marginal effects of ability to chew hard food, change in ability to chew hard food and co-variates on the outcomes (n = 1,596). MNA-SF = Mini Nutritional Assessment-Short Form; ref = Reference group; dy/dx = average difference in outcome probability (expressed in percentage points [pp]) between a category and its reference category in a variable. * Not measurable as none of the four cases who moved from co-living to an institution had a weight loss of >10% (no comparison to the reference weight loss ≤10%). Therefore, n = 1,592 in Fig. 2b (Outcome = Weight loss >10%).

Fig. 2a shows the probability of the outcomes with chewing ability at baseline as the exposure. Being in the oldest age group (80+ years old at baseline) had the greatest effect on being at risk or malnourished (assessed by MNA-SF), with the probability increased by about 17 pp (compared to being 60−69 years old). Having chewing problems at baseline increased the probability by about 6 pp (compared to having no chewing problem). In the model with the percentage weight loss outcome, chewing problems increased the probability of having more than 10% weight loss less than half of being 80+ years old at baseline (about 6 pp versus about 13 pp). Education did not affect the probability significantly, while being a woman (compared to being a man) increased probability by about 4 pp and 5pp for the MNA-SF and percentage weight loss outcomes. Living at home with someone (compared to living at home alone) decreased the probability of being at risk or malnourished, as well as having weight loss of more than 10% by about 4 pp.

Fig. 2b shows the probability of the outcomes with change in chewing ability as the exposure. Having persistent poor chewing ability or deteriorated chewing ability (compared to having no problem chewing hard food or having consistently good ability to chew hard food) increased the probabilities of having the outcomes by about 8 pp for MNA-SF at follow-up and 6 pp for >10% weight loss outcome. Individuals who moved from living alone at home to an institution (compared to continuing living alone at home) had the highest increased probabilities (about 36 and 38 pp, respectively). However, changing from co-living to an institution did not increase the probability of being at risk or malnourished at follow-up. The average marginal effects of all four models are shown in Supplementary Tables S9–S12.

## 4 Discussion

### 4.1 Summary of main results

In this longitudinal study, MNA-SF and percentage weight loss were used to assess nutritional status at the six-year follow-up. We found that self-reported difficulty chewing hard food increased the likelihood of being at risk or malnourished (measured by MNA-SF) or malnourished (measured by weight loss of >10%) at the six-year follow-up. Furthermore, individuals who had persistent difficulties chewing hard food or who lost the ability during the follow-up period were more likely to be at risk or malnourished, compared to those who never experienced such difficulty. The increased probability of being at risk or malnourished at the follow-up was small when compared to age. The increased probability of malnutrition was highest for age, followed by chewing problems, with the smallest increase associated with sex, while living with someone decreased the probability.

### 4.2 Interpretation of results

In the current study, at the six-year follow-up, 12.4% of the study population were at risk of malnutrition while 0.9% were malnourished, as measured by the MNA-SF. This was considerably lower than the pooled prevalence of 23.4% being at risk and 2.1% being malnourished found in a meta-analysis of 33 European studies using the full version of Mini Nutritional Assessment (MNA) [22]. The lower prevalence of malnutrition observed in our study compared to the meta-analysis using full MNA assessment can partly be explained by the differences between the assessment tools. MNA-SF is a screening tool and is less comprehensive than the full MNA in assessing the nutritional status. Moreover, majority of individuals included in our study were younger (only 13.2% aged 80+ years) than those in the meta-analysis study (mean age of 80+) [22]. In addition, since individuals at risk or malnourished at baseline were excluded, the samples included in this study might have been healthier than other individuals of similar age. Some variables also indicated that our population might be healthier than other populations of comparable age, i.e. the low prevalence of dysphagia (0.1%) and malabsorption and gastrointestinal diseases (0.5%; Table 1). Systematic review studies report much higher prevalence (13%–40%) of dysphagia in community dwelling older individuals [[23], [24], [25], [26]] as well as the pooled gastrointestinal disease prevalence of 31.9% in global older populations aged 65+ years [27]. In regard to mental and physical function, only 0.1% had mild to moderate dementia, while 0.1% had severe depression. All could get into and out of bed or chair and could move inside and outside the house independently. Considering the differences in sample characteristics between our study and previous studies, the prevalences in our study, as measured by MNA-SF and percentage weight loss, are not surprising.

This study used self-reported ability to chew hard food as the exposure. While a few studies have found a correlation between subjective and objective chewing ability assessments in older adults [28,29], others have either found inconsistent or contradictory associations between subjectively or objectively assessed chewing ability and health outcomes [[30], [31], [32]]. However, studies have shown that there are associations between either subjective or objective measures of chewing function with general health. Although what one perceives about the chewing function may not be equal to what one can perform, the objective assessments of chewing ability are rarely used in clinical practice due to complexity, time and resource constraints, and lack of standardization [33]. It is suggested that longitudinal studies would benefit from incorporating objective measures of chewing function, though these can often be challenging to implement in practice.

Contrary to the previous systematic review on subjective chewing ability with a one to two-year follow-up [7], the current study found that individuals with poor chewing ability were more likely to be at risk, or malnourished, at the six-year follow-up than those without self-reported chewing problems (OR = 1.64 for the MNA-SF outcome, and OR = 1.72 for the weight loss >10% outcome). One possible explanation for the contradictory findings is a difference in outcome definitions. In this study, the MNA-SF outcome in the adjusted logistic regressions included both being at risk and being malnourished at the six-year follow-up, whereas none of the community-dwelling studies in the systematic review used the MNA short-form or full-form [7]. The weight loss outcome was here defined as greater than 10% over six years. Other studies have used considerably shorter follow-up on weight loss (one year or less). Consistent with our hypothesis, the long follow-up period could have contributed to the significant association. However, additional research with similar or longer follow-up periods would be needed to confirm and generalize these findings.

Since chewing ability can change over the six-year follow-up time, additional analyses were performed using changes in chewing ability and change in changeable covariates to further explain the outcomes. The analyses show that individuals whose chewing ability deteriorated or remained poor had a higher likelihood of being at risk or malnourished at follow-up than those with normal chewing ability (OR = 1.87 for MNA-SF outcome, and OR = 1.73 for weight loss >10% outcome). These findings support our prior findings, using chewing ability at baseline as an exposure, that self-reported poor chewing ability contributes to the risk of malnutrition. This is also in line with our previous systematic review of objectively assessed chewing ability and various objectively measured nutritional parameters [12].

The average marginal effect analyses further compare the effect of chewing ability and covariates on the outcomes. Chewing ability had a relatively small effect on outcome probability when compared to being 80+ years old (about 6 pp versus about 13–17 pp, Fig. 2a). Living at home with someone reduced the probability of the two outcomes by about 4 pp compared to living alone. The models with a change in chewing ability as the exposure (Fig. 2b), also confirm that the effect of chewing ability is relatively limited. To our knowledge, no study has compared the effect of chewing ability with other malnutrition risk factors; thus, we are unable to compare our findings to those of previous studies. However, the consistent findings from the four different analyses, i.e. two exposures and two outcome measurements, indicate that the contribution from poor chewing ability to being at risk or malnourished should not be overlooked.

The results also showed that older adults who transition from living alone to an institutional setting are significantly more likely to be at risk of malnutrition or be malnourished (about a 36- to 38-pp increase compared to those who continue living alone). In contrast, moving from a co-living arrangement at home to an institution does not have a significant impact on the malnutrition risk. Additionally, those who continue co-living at home experience a 5-pp lower risk of being at risk or malnourished compared to those living alone. Poor functional status was found to be a predictor of older Swedish individuals moving to nursing homes [34], and a decline in physical function was also associated with a risk for malnutrition [35]. Based on these findings, it can be hypothesized that individuals transitioning from living alone to an institution might be in poorer health and more likely to be malnourished to begin with, when compared to those transitioning from co-living at home to an institution.

### 4.3 Strengths and limitations

This study’s strengths include its data collection methods, and the longitudinal approach used during the SNAC-K surveys, as well as the use of a secondary outcome to supplement the findings from the primary outcome.

Data at baseline and follow-up were obtained from a medical examination, a nurse interview, and the ICD-10 from the patient registry [16]. To avoid missing or poor-quality data, a registered nurse explained the options for the variables not obtained through the medical examination, such as self-reported chewing ability.

MNA-SF is a screening tool for being at risk and being malnourished. Since being "At risk of malnutrition" and "Malnourished" as assessed by MNA-SF (primary outcome) were combined due to low frequency, weight loss of >10% was chosen as the secondary outcome to measure malnutrition. The use of percentage weight loss to diagnose malnutrition is well established [36]. The cut-off of >5% over the last three months covers weight loss caused by acute illness, while the cut-off of >10% regardless of time covers both acute and chronic conditions [37]. Therefore, weight loss >10% as the secondary outcome covers a broader outcome.

The study’s limitations include a high attrition, demographic characteristics of the study population, cohort bias, the use of self-reported weight and height in some individuals, and a limited number of covariates included in the regression analyses.

First, as with any long longitudinal study in an older population, a significant number of participants were lost by the end of the six-year follow-up period. The most common reason for exclusion was non-participation in the follow-up (35.6%, Fig. 1), with more than half of those being deceased, particularly among the older participants [38]. After excluding non-participants, 13.8% were excluded due to being at risk or malnourished as assessed by MNA-SF at baseline, and missing chewing ability at baseline and follow-up, missing outcomes, or missing covariates at baseline and follow-up. The large attrition due to death could result in survival bias. A higher proportion of the individuals included in the analyses were younger, were men, had a higher education, lived with someone, had no difficulty chewing hard food and had normal nutritional status as assessed by MNA-SF (Supplementary Table S3). The survival bias could result in a healthier population with lower prevalence of malnutrition, as already pointed out. In addition, the outcomes were assessed only at the baseline and the end of the six-year follow-up, and the situations in between were unknown, such as being hospitalized during the follow-up [39,40] which might affect the outcomes. Second, individuals included in this study lived in the centre of Stockholm. Although health disparities in Sweden have decreased, there were still significant differences between municipalities in 2019 [41]. Third, the baseline period of 2002–2004 could also result in a birth cohort effect. Older individuals born in recent years may be exposed to different risk and protective factors, such as a better understanding of the causes of malnutrition, a better health-care system, or a shift toward a more or less healthy lifestyle over time. Fourth, weight and height were self-reported in 462 of the 3,363 individuals who participated in the same SNAC-K baseline survey year (2001–2004) [42]. However, a sub-sample's BMI calculated by self-reported weight and height had a statistically high correlation (Pearson correlation coefficient's r = 0.95, p-value <0.001) with the BMI calculated by measured weight and height. In addition, since the cutoff for malnutrition is more than 10% weight loss, only highly inaccurate self-reported weight could affect malnutrition prevalence. Finally, malnutrition risk factors are multifactorial; however, the covariates were limited to factors that were associated with both the exposure (chewing ability at baseline) and the main outcome (MNA-SF at follow-up). Furthermore, covariates that are part of MNA-SF were not included. Chronic diseases with systemic inflammation are other risk factors not included as covariates. Chronic negative energy balance combined with systemic inflammation can lead to changes in body composition, resulting in chronic malnutrition [43]. Indicators of systemic inflammation such as C-reactive protein (CRP) and interleukin-6 (IL-6) [44] were not included as covariates in the regression models since they are not known to be associated with chewing problems. Other risk factors for malnutrition in older people living in the community include reduced physical activity, smoking, and alcohol consumption [39,45]. Malnutrition is associated with skeletal muscle mass loss, which can affect physical activity and performance [2], but there is no known link between chewing ability and physical activity. Considering these limitations, applying the findings from our samples to other older populations in Sweden or elsewhere should be approached with caution.

## 5 Conclusion

The findings of this study suggest that poor chewing ability may be a low-risk factor for malnutrition in older individuals at the end of a long follow-up period. The results of the study emphasize the importance of poor chewing ability for the development or prevalence of malnutrition. Although the findings have limited applicability to other populations, the consistent significant relationship between two exposures and two outcomes suggests that any self-reported difficulty to chew hard food during a dental visit should be taken into consideration.

## CRediT authorship contribution statement

DL: Contributed to concept and design, acquisition of data, interpretation of data, writing the draft, revising the manuscript, and approval of the version to be submitted.

AK: Contributed to concept and design, interpretation of data, writing the draft, revising the manuscript, and approval of the version to be submitted.

SF: Performed statistical analyses, interpretation of data, writing the draft, revising the manuscript, and approval of the version to be submitted.

WX: Contributed to acquisition of data, concept and design, interpretation of data, writing the draft, revising the manuscript, and approval of the version to be submitted.

GSE: Concept and design, interpretation of data, writing the draft, revising the manuscript, and approval of the version to be submitted.

## Funding

This research did not receive any specific grant from funding agencies in the public, commercial, or not-for-profit sectors.

## Data availability statement

The author group obtained the data from the SNAC-K study and the data are not open to access beyond this study.

## Declaration of competing interest

Duangjai Lexomboon reports administrative support, article publishing charges, statistical analysis, and writing assistance were provided by Karolinska Institute. If there are other authors, they declare that they have no known competing financial interests or personal relationships that could have appeared to influence the work reported in this paper.
