# Supplementary material for: Is poor chewing ability a risk factor for malnutrition? A six-year longitudinal study of older adults in Sweden
Source: J Nutr Health Aging. 2025 Apr 2;29(6):100554. doi: 10.1016/j.jnha.2025.100554 (PMC12173007; doi:10.1016/j.jnha.2025.100554)
Supplement: Supplementary file 1 [file mmc1.docx]

**Supplemental materials**

Supplementary Table 1. Definitions of different categories for change in ability to chew hard food and change in living arrangement.

Supplementary Table 2. Univariate analysis between co-variates and Body Mass Index and the main outcome (MNA-SF at follow-up).

Supplementary Table S3. Characteristics of individuals included and excluded from the analysis.

Supplementary Table S4. Characteristics of individuals who had change in ability to chew hard food.

Supplementary Table S5. Logistic regression model: ability to chew hard food at baseline as the exposure and MNA-SF at follow-up as the outcome, crude and adjusted for co-variates.

Supplementary Table S6. Logistic regression model: ability to chew hard food at baseline as the exposure and weight loss >10% as the outcome, crude, and adjusted models.

Supplementary Table S7. Logistic regression model: change in ability to chew hard food as the exposure and MNA-SF at follow-up as the outcome, crude, and adjusted models.

Supplementary Table S8. Logistic regression model: change in ability to chew hard food as the exposure and weight loss >10% as the outcome, crude and adjusted for co-variates.

Supplementary Table S9. Average marginal effects: ability to chew hard food at baseline as the exposure and MNA-SF at follow-up as the outcome.

Supplementary Table S10. Average marginal effects: ability to chew hard food at baseline as the exposure and weight loss >10% as the outcome.

Supplementary Table S11. Average marginal effects: change in ability to chew hard food as the exposure and MNA-SF at follow-up as the outcome.

Supplementary Table S12. Average marginal effects: change in ability to chew hard food as the exposure and weight loss >10% as the outcome.

**Supplementary Table 1. Definitions and prevalence rates of different categories for change in ability to chew hard food and change in living arrangement.**

| **Change in ability to chew hard food** | **Definition** | **n (%)** |
| --- | --- | --- |
| Consistently good | No problem at both baseline and follow-up | 1319 (82.6%) |
| Improved | Had a problem at baseline but no problem at follow-up | 86 (5.4%) |
| Declined | No problem at baseline but developed a problem at follow-up | 127 (8%) |
| Persistent problem | Had a problem at both baseline and follow-up | 64 (4%) |
| **Change in living arrangement** | **Definition** | **n (%)** |
| Living alone | Living alone at home at both baseline and follow-up | 658 (41.2%) |
| Co-living | Living with someone at home at both baseline and follow-up | 746 (46.7%) |
| Transition to co-living | Changed from living alone at home to living with someone at home | 62 (3.9%) |
| Transition to living alone | Changed from living with someone at home to living alone at home | 110 (6.9%) |
| Living alone to institution | Changed from living alone at home to living in an institution | 16 (1%) |
| Co-living to institution | Changed from living with someone at home to living in an institution | 4 (0.3%) |

**Supplementary Table 2. Univariate analysis between co-variates and Body Mass Index at baseline and the main outcome (MNA-SF at follow-up).**

|  |  |  |  |  | **MNA-SF at follow-up, n (%)** | | | |
| --- | --- | --- | --- | --- | --- | --- | --- | --- |
|  |  |  | **Total** |  | **Normal** | **At risk** | **Malnourished** | **p-value** |
| **Total n (%) *** |  |  | 1,596 |  | 1,384 (86.7%) | 198 (12.4%) | 14 (0.9%) |  |
| **Age (year)** | 60-69 |  | 893 (56%) |  | 814 (58.8%) | 78 (39.4%) | 1 (7.1%) | <0.001 |
|  | 70-79 |  | 492 (30.8%) |  | 420 (30.3%) | 64 (32.3%) | 8 (57.1%) |  |
|  | 80+ |  | 211 (13.2%) |  | 150 (10.8%) | 56 (28.3%) | 5 (35.7%) |  |
| **Sex** | Men |  | 615 (38.5%) |  | 557 (40.2%) | 55 (27.8%) | 3 (21.4%) | 0.001 |
|  | Women |  | 981 (61.5%) |  | 827 (59.8%) | 143 (72.2%) | 11 (78.6%) |  |
| **Education** | Elementary school |  | 310 (19.4%) |  | 254 (18.4%) | 49 (24.7%) | 7 (50%) | 0.002 |
|  | High school |  | 683 (42.8%) |  | 593 (42.8%) | 83 (41.9%) | 7 (50%) |  |
|  | University |  | 603 (37.8%) |  | 537 (38.8%) | 66 (33.3%) | 0 (0%) |  |
| **Living arrangement** | Living alone |  | 736 (46.1%) |  | 607 (43.9%) | 119 (60.1%) | 10 (71.4%) | <0.001 |
|  | With someone |  | 860 (53.9%) |  | 777 (56.1%) | 79 (39.9%) | 4 (28.6%) |  |
| **Xerostomia** | No |  | 1578 (98.9%) |  | 1368 (98.8%) | 197 (99.5%) | 13 (92.9%) | 0.073 |
|  | Yes |  | 18 (1.1%) |  | 16 (1.2%) | 1 (0.5%) | 1 (7.1%) |  |
| **Dysphagia** | No |  | 1595 (99.9%) |  | 1384 (100%) | 197 (99.5%) | 14 (100%) | 0.029 |
|  | Yes |  | 1 (0.1%) |  | 0 (0%) | 1 (0.5%) | 0 (0%) |  |
| **Malabsorption & GI diseases** | No |  | 1588 (99.5%) |  | 1376 (99.4%) | 198 (100%) | 14 (100%) | 0.540 |
|  | Yes |  | 8 (0.5%) |  | 8 (0.6%) | 0 (0%) | 0 (0%) |  |
| **Cancer** | No |  | 1513 (94.8%) |  | 1316 (95.1%) | 183 (92.4%) | 14 (100%) | 0.195 |
|  | Yes |  | 83 (5.2%) |  | 68 (4.9%) | 15 (7.6%) | 0 (0%) |  |
| **BMI at baseline** | Mean±SD |  | 26.3±3.7 |  | 26.5±3.7 | 24.8±3.6 | 25.6±5.1 | <0.001 |

MNA-SF = Mini Nutritional Assessment-Short Form; BMI = Body Mass Index; GI diseases = Gastrointestinal diseases

* Percent row

**Supplementary Table S3. Characteristics of individuals included and excluded from the analysis.**

|  |  | Total  n=3,155 | Included  n=1,596 | Excluded  n=1,559 | p-value |
| --- | --- | --- | --- | --- | --- |
| Age (year) | 60-69 | 1298 (41.1%) | 893 (56%) | 405 (26%) | <0.001 |
|  | 70-79 | 914 (29%) | 492 (30.8%) | 422 (27.1%) |  |
|  | 80+ | 943 (29.9%) | 211 (13.2%) | 732 (47%) |  |
| Sex | Men | 1149 (36.4%) | 615 (38.5%) | 534 (34.3%) | 0.012 |
|  | Women | 2006 (63.6%) | 981 (61.5%) | 1025 (65.7%) |  |
| Education | Elementary school | 838 (26.6%) | 310 (19.4%) | 528 (33.9%) | <0.001 |
|  | High school | 1332 (42.2%) | 683 (42.8%) | 649 (41.6%) |  |
|  | University | 980 (31.1%) | 603 (37.8%) | 377 (24.2%) |  |
|  | Missing | 5 (0.2%) | 0 (0%) | 5 (0.3%) |  |
| Living arrangement | Alone | 1771 (56.1%) | 736 (46.1%) | 1035 (66.4%) | <0.001 |
|  | With someone | 1384 (43.9%) | 860 (53.9%) | 524 (33.6%) |  |
| Xerostomia | No | 3108 (98.5%) | 1578 (98.9%) | 1530 (98.1%) | 0.090 |
|  | Yes | 47 (1.5%) | 18 (1.1%) | 29 (1.9%) |  |
| Dysphagia | No | 3154 (100%) | 1595 (99.9%) | 1559 (100%) | 0.323 |
|  | Yes | 1 (0%) | 1 (0.1%) | 0 (0%) |  |
| Malabsorption & GI diseases | No | 3146 (99.7%) | 1588 (99.5%) | 1558 (99.9%) | 0.021 |
|  | Yes | 9 (0.3%) | 8 (0.5%) | 1 (0.1%) |  |
| Cancer | No | 2979 (94.4%) | 1513 (94.8%) | 1466 (94%) | 0.349 |
|  | Yes | 176 (5.6%) | 83 (5.2%) | 93 (6%) |  |
| Ability to chew hard food at baseline | No problem | 2678 (84.9%) | 1446 (90.6%) | 1232 (79%) | <0.001 |
|  | Must be careful | 296 (9.4%) | 88 (5.5%) | 208 (13.3%) |  |
|  | Cannot at all | 164 (5.2%) | 62 (3.9%) | 102 (6.5%) |  |
|  | Missing | 17 (0.5%) | 0 (0%) | 17 (1.1%) |  |
| BMI at baseline | Mean±SD | 29.9±17.6 | 26.3±3.7 | 33.5±24.2 | <0.001 |
| MNA-SF at baseline | Normal | 2429 (77%) | 1596 (100%) | 833 (53.4%) | <0.001 |
|  | At risk | 433 (13.7%) | 0 (0%) | 433 (27.8%) |  |
|  | Malnourished | 18 (0.6%) | 0 (0%) | 18 (1.2%) |  |
|  | Missing | 275 (8.7%) | 0 (0%) | 275 (17.6%) |  |

MNA-SF = Mini Nutritional Assessment-Short Form; BMI = Body Mass Index; GI = Gastrointestinal diseases

**Supplementary Table S4. Characteristics of individuals who had change in ability to chew hard food.**

|  |  |  | **Change in ability to chew hard food, n (%)** | | | |  |
| --- | --- | --- | --- | --- | --- | --- | --- |
|  |  | **Total** | Consistently good | Improved | Declined/persistent problem | **p-value** | |
| **Total n (%) *** |  | 1,596 | 1,319 (82.6%) | 86 (5.4%) | 191 (12.0%) |  | |
| **Age (year)** | 60-69 | 893 (56%) | 794 (60.2%) | 37 (43%) | 62 (32.5%) | <0.001 | |
|  | 70-79 | 492 (30.8%) | 377 (28.6%) | 35 (40.7%) | 80 (41.9%) |  | |
|  | 80+ | 211 (13.2%) | 148 (11.2%) | 14 (16.3%) | 49 (25.7%) |  | |
| **Sex** | Men | 615 (38.5%) | 527 (40%) | 31 (36%) | 57 (29.8%) | 0.024 | |
|  | Women | 981 (61.5%) | 792 (60%) | 55 (64%) | 134 (70.2%) |  | |
| **Education** | Elementary school | 310 (19.4%) | 225 (17.1%) | 26 (30.2%) | 59 (30.9%) | <0.001 | |
|  | High school | 683 (42.8%) | 558 (42.3%) | 42 (48.8%) | 83 (43.5%) |  | |
|  | University | 603 (37.8%) | 536 (40.6%) | 18 (20.9%) | 49 (25.7%) |  | |
| **Change in living arrangement** | Living alone | 658 (41.2%) | 518 (39.3%) | 46 (53.5%) | 94 (49.2%) | <0.001 | |
|  | Co-living | 746 (46.7%) | 645 (48.9%) | 29 (33.7%) | 72 (37.7%) |  | |
|  | Transition to co-living | 62 (3.9%) | 55 (4.2%) | 3 (3.5%) | 4 (2.1%) |  | |
|  | Transition to living alone | 110 (6.9%) | 89 (6.7%) | 8 (9.3%) | 13 (6.8%) |  | |
|  | Living alone to institution | 16 (1.0%) | 10 (0.8%) | 0 (0%) | 6 (3.1%) |  | |
|  | Co-living to institution | 4 (0.3%) | 2 (0.2%) | 0 (0%) | 2 (1%) |  | |
| **BMI at baseline** | Mean±SD | 26.3±3.7 | 26.3±3.7 | 26.8±3.8 | 26.1±3.7 | 0.314 | |

BMI = Body Mass Index

* Percent row

**Supplementary Table S5. Logistic regression model: ability to chew hard food at baseline as the exposure and MNA-SF as the outcome, crude and adjusted models.**

| **Logistic regression: Crude model** | | | Number of observations | | | = | 1,596 | |
| --- | --- | --- | --- | --- | --- | --- | --- | --- |
|  |  |  | Wald chi2 (1) | | | = | 12.19 | |
|  |  |  | Probability > chi2 | | | = | 0.0005 | |
| Log pseudolikelihood = -619.71696 | | | Pseudo R2 | | | = | 0.0088 | |
|  |  | Robust |  |  |  | | |  |
| MNA-SF at follow-up | Odds ratio | Std. Err. | z | P>\|Z\| | [95% CI] | | | |
| Ability to chew hard food at baseline |  |  |  |  |  | | |  |
| No problem | 1 | (base) |  |  |  | | |  |
| Cannot/Must be careful | 2.087950 | 0.440290 | 3.49 | 0.000 | 1.381102 | | | 3.156564 |
|  |  |  |  |  |  | | |  |
| _cons | 0.140379 | 0.011240 | -24.52 | 0.000 | 0.119991 | | | 0.164230 |

| **Logistic regression: Adjusted model** | | | Number of observations | | | = | 1,596 | |
| --- | --- | --- | --- | --- | --- | --- | --- | --- |
|  |  |  | Wald chi2 (8) | | | = | 77.95 | |
|  |  |  | Probability > chi2 | | | = | 0.0000 | |
| Log pseudolikelihood = -587.47001 | | | Pseudo R2 | | | = | 0.0604 | |
|  |  | Robust |  |  |  | | |  |
| MNA-SF at follow-up | Odds ratio | Std. Err. | z | P>\|Z\| | [95% CI] | | | |
| Ability to chew hard food at baseline |  |  |  |  |  | | |  |
| No problem | 1 | (base) |  |  |  | | |  |
| Cannot/Must be careful | 1.637335 | 0.362705 | 2.23 | 0.026 | 1.060664 | | | 2.527535 |
|  |  |  |  |  |  | | |  |
| Age (year) |  |  |  |  |  | | |  |
| 60-69 | 1 | (base) |  |  |  | | |  |
| 70-79 | 1.599323 | 0.280373 | 2.68 | 0.007 | 1.134264 | | | 2.255061 |
| ≥80 | 3.483374 | 0.722678 | 6.02 | 0.000 | 2.319560 | | | 5.231120 |
|  |  |  |  |  |  | | |  |
| Sex |  |  |  |  |  | | |  |
| Man | 1 | (base) |  |  |  | | |  |
| Woman | 1.461309 | 0.260014 | 2.13 | 0.033 | 1.031064 | | | 2.071087 |
|  |  |  |  |  |  | | |  |
| Education |  |  |  |  |  | | |  |
| Elementary school | 1 | (base) |  |  |  | | |  |
| High school | 0.855952 | 0.169859 | -0.78 | 0.433 | 0.580141 | | | 1.262889 |
| University | 0.927470 | 0.202885 | -0.34 | 0.731 | 0.604086 | | | 1.423971 |
|  |  |  |  |  |  | | |  |
| Living arrangement |  |  |  |  |  | | |  |
| At home alone | 1 | (base) |  |  |  | | |  |
| At home with someone | 0.665522 | 0.110139 | -2.46 | 0.014 | 0.481166 | | | 0.920514 |
|  |  |  |  |  |  | | |  |
| _cons | 0.101560 | 0.026631 | -8.72 | 0.000 | 0.060746 | | | 0.169796 |

_cons = Estimated baseline odds

**Supplementary Table S6. Logistic regression model: ability to chew hard food at baseline as the exposure and Weight loss >10%** **as the outcome, crude and adjusted for co-variates.**

| **Logistic regression: Crude model** | | | Number of observations | | | = | 1.596 | |
| --- | --- | --- | --- | --- | --- | --- | --- | --- |
|  |  |  | Wald chi2 (1) | | | = | 14.16 | |
|  |  |  | Probability > chi2 | | | = | 0.0002 | |
| Log pseudolikelihood = -553.92243 | | | Pseudo R2 | | | = | 0.0112 | |
|  |  | Robust |  |  |  | | |  |
| Weight loss >10% | Odds ratio | Std. Err. | z | P>\|Z\| | [95% CI] | | | |
| Ability to chew hard food at baseline |  |  |  |  |  | | |  |
| No problem | 1 | (base) |  |  |  | | |  |
| Cannot/Must be careful | 2.284692 | 0.50169 | 3.76 | 0.000 | 1.485642 | | | 3.513510 |
|  |  |  |  |  |  | | |  |
| _cons | 0.114022 | 0.00990 | -25.02 | 0.000 | 0.096187 | | | 0.135164 |

| **Logistic regression: Adjusted model** | | | Number of observations | | | = | 1,596 | |
| --- | --- | --- | --- | --- | --- | --- | --- | --- |
|  |  |  | Wald chi2 (7) | | | = | 81.31 | |
|  |  |  | Probability > chi2 | | | = | 0.0000 | |
| Log pseudolikelihood = -519.2588 | | | Pseudo R2 | | | = | 0.0731 | |
|  |  | Robust |  |  |  | | |  |
| Weight loss >10% | Odds ratio | Std. Err. | z | P>\|Z\| | [95% CI] | | | |
| Ability to chew hard food at baseline |  |  |  |  |  | | |  |
| No problem | 1 | (base) |  |  |  | | |  |
| Cannot/Must be careful | 1.717035 | 0.390538 | 2.38 | 0.017 | 1.099446 | | | 2.681540 |
|  |  |  |  |  |  | | |  |
| Age (year) |  |  |  |  |  | | |  |
| 60-69 | 1 | (base) |  |  |  | | |  |
| 70-79 | 2.366785 | 0.453123 | 4.5 | 0.000 | 1.626282 | | | 3.444466 |
| ≥80 | 3.545875 | 0.826117 | 5.43 | 0.000 | 2.246005 | | | 5.598043 |
|  |  |  |  |  |  | | |  |
| Sex |  |  |  |  |  | | |  |
| Man | 1 | (base) |  |  |  | | |  |
| Woman | 1.723319 | 0.344257 | 2.72 | 0.006 | 1.165001 | | | 2.549207 |
|  |  |  |  |  |  | | |  |
| Education |  |  |  |  |  | | |  |
| Elementary school | 1 | (base) |  |  |  | | |  |
| High school | 1.065846 | 0.226495 | 0.3 | 0.764 | 0.702768 | | | 1.616504 |
| University | 0.897885 | 0.220122 | -0.44 | 0.660 | 0.555320 | | | 1.451770 |
|  |  |  |  |  |  | | |  |
| Living arrangement |  |  |  |  |  | | |  |
| At home alone | 1 | (base) |  |  |  | | |  |
| At home with someone | 0.685464 | 0.125758 | -2.06 | 0.040 | 0.478431 | | | 0.982086 |
|  |  |  |  |  |  | | |  |
| _cons | 0.056600 | 0.017206 | -9.45 | 0.000 | 0.031193 | | | 0.102702 |

_cons = Estimates baseline odds; MNA-SF = Mini Nutritional Assessment-Short Form

**Supplementary Table S7. Logistic regression model: change in ability to chew hard food as the exposure and MNA-SF at follow-up as the outcome, crude, and adjusted models.**

| **Logistic regression: Crude model** | | | Number of observations | | | = | 1,596 | |
| --- | --- | --- | --- | --- | --- | --- | --- | --- |
|  |  |  | Wald chi2 (2) | | | = | 27.84 | |
|  |  |  | Probability > chi2 | | | = | 0.0000 | |
| Log pseudolikelihood = -612.43525 | | | Pseudo R2 | | | = | 0.0204 | |
|  |  | Robust |  |  |  | | |  |
| MNA-SF at follow-up | Odds ratio | Std. Err. | z | P>\|Z\| | [95% CI] | | | |
| Change in ability to chew hard food |  |  |  |  |  | | |  |
| Good consistently | 1 | (base) |  |  |  | | |  |
| Improved | 1.949373 | 0.554725 | 2.35 | 0.019 | 1.11602 | | | 3.405006 |
| Declined or persistent problem | 2.582442 | 0.488991 | 5.01 | 0.000 | 1.781778 | | | 3.742893 |
|  |  |  |  |  |  | | |  |
| _cons | 0.126388 | 0.01103 | -23.7 | 0.000 | 0.106518 | | | 0.149964 |

| **Logistic regression: Adjusted model** | | | Number of observations | | | = | 1,596 | |
| --- | --- | --- | --- | --- | --- | --- | --- | --- |
|  |  |  | Wald chi2 (7) | | | = | 96.47 | |
|  |  |  | Probability > chi2 | | | = | 0.0000 | |
| Log pseudolikelihood = -575.91911 | | | Pseudo R2 | | | = | 0.0788 | |
|  |  | Robust |  |  |  | | |  |
| MNA-SF at follow-up | Odds ratio | Std. Err. | z | P>\|Z\| | [95% CI] | | | |
| Change in ability to chew hard food |  |  |  |  |  | | |  |
| Good consistently | 1 | (base) |  |  |  | | |  |
| Improved | 1.740197 | 0.512142 | 1.88 | 0.060 | 0.977437 | | | 3.098189 |
| Declined or persistent problem | 1.874341 | 0.379641 | 3.10 | 0.002 | 1.260204 | | | 2.787766 |
|  |  |  |  |  |  | | |  |
| Age (year) |  |  |  |  |  | | |  |
| 60-69 | 1 | (base) |  |  |  | | |  |
| 70-79 | 1.463744 | 0.260083 | 2.14 | 0.032 | 1.033286 | | | 2.073525 |
| ≥80 | 2.783738 | 0.615704 | 4.63 | 0.000 | 1.804514 | | | 4.294338 |
|  |  |  |  |  |  | | |  |
| Sex |  |  |  |  |  | | |  |
| Man | 1 | (base) |  |  |  | | |  |
| Woman | 1.383596 | 0.253373 | 1.77 | 0.076 | 0.966344 | | | 1.981011 |
|  |  |  |  |  |  | | |  |
| Education |  |  |  |  |  | | |  |
| Elementary school | 1 | (base) |  |  |  | | |  |
| High school | 0.895167 | 0.180614 | -0.55 | 0.583 | 0.602782 | | | 1.329375 |
| University | 0.966471 | 0.2156 | -0.15 | 0.878 | 0.62417 | | | 1.496493 |
|  |  |  |  |  |  | | |  |
| Change in living arrangement |  |  |  |  |  | | |  |
| Living alone | 1 | (base) |  |  |  | | |  |
| Co-living | 0.620854 | 0.115606 | -2.56 | 0.010 | 0.431014 | | | 0.894307 |
| Transition to co-living | 0.739839 | 0.339081 | -0.66 | 0.511 | 0.301312 | | | 1.816593 |
| Transition to living alone | 0.883891 | 0.264144 | -0.41 | 0.680 | 0.492066 | | | 1.587720 |
| Living alone to institution | 6.462788 | 3.985987 | 3.03 | 0.002 | 1.929448 | | | 21.647450 |
| Co-living to institution | 3.646545 | 4.025308 | 1.17 | 0.241 | 0.419051 | | | 31.731940 |
|  |  |  |  |  |  | | |  |
| _cons | 0.099285 | 0.027286 | -8.40 | 0.000 | 0.057937 | | | 0.170143 |

_cons = Estimated baseline odds; MNA-SF = Mini Nutritional Assessment-Short Form

**Supplementary Table S8. Logistic regression model: change in ability to chew hard food as the exposure and weight loss >10% as the outcome, crude and adjusted for co-variates.**

| **Logistic regression: Crude model** | | | Number of observations | | | = | 1,592* | |
| --- | --- | --- | --- | --- | --- | --- | --- | --- |
|  |  |  | Wald chi2 (2) | | | = | 20.79 | |
|  |  |  | Probability > chi2 | | | = | 0.0000 | |
| Log pseudolikelihood = -550.37873 | | | Pseudo R2 | | | = | 0.0167 | |
|  |  | Robust |  |  |  | | |  |
| Weight loss >10% | Odds ratio | Std. Err. | z | P>\|Z\| | [95% CI] | | | |
| Change in ability to chew hard food |  |  |  |  |  | | |  |
| Good consistently | 1 | (base) |  |  |  | | |  |
| Improved | 1.362396 | 0.457909 | 0.92 | 0.358 | 0.705034 | | | 2.632673 |
| Declined or persistent problem | 2.493708 | 0.501169 | 4.55 | 0.000 | 1.681809 | | | 3.697554 |
|  |  |  |  |  |  | | |  |
| _cons | 0.107654 | 0.010018 | -23.95 | 0.000 | 0.089706 | | | 0.129192 |

| **Logistic regression: Adjusted model** | | | Number of observations | | | = | 1,592* | |
| --- | --- | --- | --- | --- | --- | --- | --- | --- |
|  |  |  | Wald chi2 (7) | | | = | 91.14 | |
|  |  |  | Probability > chi2 | | | = | 0.0000 | |
| Log pseudolikelihood = -509.848 | | | Pseudo R2 | | | = | 0.0891 | |
|  |  | Robust |  |  |  | | |  |
| Weight loss > 10% | Odds ratio | Std. Err. | z | P>\|Z\| | [95% CI] | | | |
| Change in ability to chew hard food |  |  |  |  |  | | |  |
| Good consistently | 1 | (base) |  |  |  | | |  |
| Improved | 1.151331 | 0.390150 | 0.42 | 0.678 | 0.592590 | | | 2.236896 |
| Declined or persistent problem | 1.727909 | 0.377897 | 2.50 | 0.012 | 1.125541 | | | 2.652652 |
|  |  |  |  |  |  | | |  |
| Age (year) |  |  |  |  |  | | |  |
| 60-69 | 1 | (base) |  |  |  | | |  |
| 70-79 | 2.235208 | 0.432039 | 4.16 | 0.000 | 1.530350 | | | 3.264712 |
| ≥80 | 2.927598 | 0.729961 | 4.31 | 0.000 | 1.795869 | | | 4.772525 |
|  |  |  |  |  |  | | |  |
| Sex |  |  |  |  |  | | |  |
| Man | 1 | (base) |  |  |  | | |  |
| Woman | 1.655995 | 0.338779 | 2.47 | 0.014 | 1.108977 | | | 2.472838 |
|  |  |  |  |  |  | | |  |
| Education |  |  |  |  |  | | |  |
| Elementary school | 1 | (base) |  |  |  | | |  |
| High school | 1.111236 | 0.242006 | 0.48 | 0.628 | 0.725155 | | | 1.702872 |
| University | 0.897031 | 0.226187 | -0.43 | 0.667 | 0.547238 | | | 1.470411 |
|  |  |  |  |  |  | | |  |
| Change in living arrangement |  |  |  |  |  | | |  |
| Living alone | 1 | (base) |  |  |  | | |  |
| Co-living | 0.692085 | 0.144503 | -1.76 | 0.078 | 0.459657 | | | 1.042040 |
| Transition to co-living | 1.187040 | 0.518533 | 0.39 | 0.695 | 0.504238 | | | 2.794439 |
| Transition to living alone | 0.960717 | 0.293072 | -0.13 | 0.895 | 0.528362 | | | 1.746863 |
| Living alone to institution | 8.498305 | 4.844821 | 3.75 | 0.000 | 2.780152 | | | 25.977420 |
| Co-living to institution | 1 | (empty) |  |  |  | | |  |
|  |  |  |  |  |  | | |  |
| _cons | 0.054683 | 0.017642 | -9.01 | 0.000 | 0.0290559 | | | 0.102913 |

_cons = Estimated baseline odds

* Four cases who moved from co-living to an institution were excluded because none had weight loss of >10% to compare to the reference (weight loss ≤10%).

**Supplementary Table S9. Average marginal effects: ability to chew hard food at baseline as the exposure and MNA-SF at follow-up as the outcome.**

| **Average marginal effects** |  |  | Number of observations | | | = | 1,596 | |
| --- | --- | --- | --- | --- | --- | --- | --- | --- |
|  |  | Delta-method |  |  |  | | |  |
| MNA-SF at follow-up | dy/dx | Std. Err. | z | P>\|Z\| | [95% CI] | | | |
| Ability to chew hard food at baseline |  |  |  |  |  | | |  |
| No problem | (Base) |  |  |  |  | | |  |
| Cannot/Must be careful | 0.061119 | 0.030838 | 1.98 | 0.047 | 0.000678 | | | 0.121561 |
|  |  |  |  |  |  | | |  |
| Age (year) |  |  |  |  |  | | |  |
| 60-69 | (Base) |  |  |  |  | | |  |
| 70-79 | 0.047397 | 0.018300 | 2.59 | 0.010 | 0.011531 | | | 0.083264 |
| ≥80 | 0.166738 | 0.033116 | 5.03 | 0.000 | 0.101832 | | | 0.231645 |
|  |  |  |  |  |  | | |  |
| Sex |  |  |  |  |  | | |  |
| Man | (Base) |  |  |  |  | | |  |
| Woman | 0.039868 | 0.018026 | 2.21 | 0.027 | 0.004538 | | | 0.075198 |
|  |  |  |  |  |  | | |  |
| Education |  |  |  |  |  | | |  |
| Elementary school | (Base) |  |  |  |  | | |  |
| High school | -0.017141 | 0.022335 | -0.77 | 0.443 | -0.060917 | | | 0.026635 |
| University | -0.008519 | 0.024905 | -0.34 | 0.732 | -0.057331 | | | 0.040294 |
|  |  |  |  |  |  | | |  |
| Living arrangement |  |  |  |  |  | | |  |
| At home alone | (Base) |  |  |  |  | | |  |
| At home with someone | -0.044462 | 0.018058 | -2.46 | 0.014 | -0.079855 | | | -0.009069 |

dy/dx = average difference in outcome probability between a category and its reference category in a variable; MNA-SF = Mini Nutritional Assessment-Short Form

**Supplementary Table S10. Average marginal effects: ability to chew hard food at baseline as the exposure and weight loss >10% as the outcome.**

| **Average marginal effects** |  |  | Number of observations | | | = | 1,596 | |
| --- | --- | --- | --- | --- | --- | --- | --- | --- |
|  |  | Delta-method |  |  |  | | |  |
| Weight loss >10% | dy/dx | Std. Err. | z | P>\|Z\| | [95% CI] | | | |
| Ability to chew hard food at baseline |  |  |  |  |  | | |  |
| No problem | (Base) |  |  |  |  | | |  |
| Cannot/Must be careful | 0.058759 | 0.028119 | 2.09 | 0.037 | 0.003646 | | | 0.113872 |
|  |  |  |  |  |  | | |  |
| Age (year) |  |  |  |  |  | | |  |
| 60-69 | (Base) |  |  |  |  | | |  |
| 70-79 | 0.077024 | 0.017869 | 4.31 | 0.000 | 0.042001 | | | 0.112047 |
| ≥80 | 0.132845 | 0.029692 | 4.47 | 0.000 | 0.074650 | | | 0.191041 |
|  |  |  |  |  |  | | |  |
| Sex |  |  |  |  |  | | |  |
| Man | (Base) |  |  |  |  | | |  |
| Woman | 0.048099 | 0.016551 | 2.91 | 0.004 | 0.015659 | | | 0.080538 |
|  |  |  |  |  |  | | |  |
| Education |  |  |  |  |  | | |  |
| Elementary school | (Base) |  |  |  |  | | |  |
| High school | 0.006158 | 0.020332 | 0.30 | 0.762 | -0.033692 | | | 0.046008 |
| University | -0.009797 | 0.022447 | -0.44 | 0.663 | -0.053791 | | | 0.034198 |
|  |  |  |  |  |  | | |  |
| Living arrangement |  |  |  |  |  | | |  |
| At home alone | (Base) |  |  |  |  | | |  |
| At home with someone | -0.035365 | 0.017164 | -2.06 | 0.039 | -0.069007 | | | -0.001724 |

dy/dx = average difference in outcome probability between a category and its reference category in a variable

**Supplementary Table S11. Average marginal effects: change in ability to chew hard food as the exposure and MNA-SF at follow-up as the outcome.**

| **Average marginal effects** |  |  | Number of observations | | | = | 1,596 | |
| --- | --- | --- | --- | --- | --- | --- | --- | --- |
|  |  | Delta-method |  |  |  | | |  |
| MNA-SF at follow-up | dy/dx | std. err. | z | P>\|Z\| | [95% CI] | | | |
| Change in ability to chew hard food |  |  |  |  |  | | |  |
| Good consistently | (Base) |  |  |  |  | | |  |
| Improved | 0.0656769 | 0.0402801 | 1.63 | 0.103 | -0.0132707 | | | 0.1446245 |
| Declined or persistent problem | 0.0763127 | 0.0281709 | 2.71 | 0.007 | 0.0210988 | | | 0.1315266 |
|  |  |  |  |  |  | | |  |
| Age (year) |  |  |  |  |  | | |  |
| 60-69 | (Base) |  |  |  |  | | |  |
| 70-79 | 0.0382894 | 0.0182699 | 2.10 | 0.036 | 0.0024810 | | | 0.0740978 |
| ≥80 | 0.1291019 | 0.0327775 | 3.94 | 0.000 | 0.0648593 | | | 0.1933446 |
|  |  |  |  |  |  | | |  |
| Sex |  |  |  |  |  | | |  |
| Man | (Base) |  |  |  |  | | |  |
| Woman | 0.0334559 | 0.0183229 | 1.83 | 0.068 | -0.0024563 | | | 0.0693681 |
|  |  |  |  |  |  | | |  |
| Education |  |  |  |  |  | | |  |
| Elementary school | (Base) |  |  |  |  | | |  |
| High school | -0.0118052 | 0.0218339 | -0.54 | 0.589 | -0.0545989 | | | 0.0309886 |
| University | -0.0037276 | 0.0244503 | -0.15 | 0.879 | -0.0516493 | | | 0.0441941 |
|  |  |  |  |  |  | | |  |
| Change in living arrangement |  |  |  |  |  | | |  |
| Living alone | (Base) |  |  |  |  | | |  |
| Co-living | -0.0500397 | 0.0193789 | -2.58 | 0.010 | -0.0880217 | | | -0.0120577 |
| Transition to co-living | -0.0335640 | 0.0466886 | -0.72 | 0.472 | -0.1250719 | | | 0.0579439 |
| Transition to living alone | -0.0145874 | 0.0342542 | -0.43 | 0.670 | -0.0817245 | | | 0.0525496 |
| Living alone to institution | 0.3596787 | 0.1458022 | 2.47 | 0.014 | 0.0739115 | | | 0.6454458 |
| Co-living to institution | 0.2270344 | 0.2445232 | 0.93 | 0.353 | -0.2522223 | | | 0.7062911 |

dy/dx = average difference in outcome probability between a category and its reference category in a variable; MNA-SF = Mini Nutritional Assessment-Short Form

**Supplementary Table S12. Average marginal effects: change in ability to chew hard food as the exposure and weight loss >10% as the outcome.**

| **Average marginal effects** |  |  | Number of observations | | | = | 1,592* | |
| --- | --- | --- | --- | --- | --- | --- | --- | --- |
|  |  | Delta-method |  |  |  | | |  |
| Weight loss >10% | dy/dx | std. err. | z | P>\|Z\| | [95% CI] | | | |
| Change in ability to chew hard food |  |  |  |  |  | | |  |
| Good consistently | (Base) |  |  |  |  | | |  |
| Improved | 0.0128174 | 0.0321403 | 0.40 | 0.690 | -0.0501765 | | | 0.0758112 |
| Declined or persistent problem | 0.0573829 | 0.0259427 | 2.21 | 0.027 | 0.0065362 | | | 0.1082297 |
|  |  |  |  |  |  | | |  |
| Age (year) |  |  |  |  |  | | |  |
| 60-69 | (Base) |  |  |  |  | | |  |
| 70-79 | 0.0720170 | 0.0178927 | 4.02 | 0.000 | 0.0369479 | | | 0.1070860 |
| ≥80 | 0.1068753 | 0.0297018 | 3.60 | 0.000 | 0.0486609 | | | 0.1650897 |
|  |  |  |  |  |  | | |  |
| Sex |  |  |  |  |  | | |  |
| Man | (Base) |  |  |  |  | | |  |
| Woman | 0.0438184 | 0.0167323 | 2.62 | 0.009 | 0.0110237 | | | 0.0766132 |
|  |  |  |  |  |  | | |  |
| Education |  |  |  |  |  | | |  |
| Elementary school | (Base) |  |  |  |  | | |  |
| High school | 0.0099703 | 0.0202548 | 0.49 | 0.623 | -0.0297284 | | | 0.0496689 |
| University | -0.0095303 | 0.0222752 | -0.43 | 0.669 | -0.0531889 | | | 0.0341284 |
|  |  |  |  |  |  | | |  |
| Change in living arrangement |  |  |  |  |  | | |  |
| Living alone | (Base) |  |  |  |  | | |  |
| Co-living | -0.0327819 | 0.0184963 | -1.77 | 0.076 | -0.0690339 | | | 0.0034702 |
| Transition to co-living | 0.0184598 | 0.0492356 | 0.37 | 0.708 | -0.0780401 | | | 0.1149597 |
| Transition to living alone | -0.0040095 | 0.0302372 | -0.13 | 0.895 | -0.0632733 | | | 0.0552542 |
| Living alone to institution | 0.3805632 | 0.1299167 | 2.93 | 0.003 | 0.1259312 | | | 0.6351952 |
| Co-living to institution | . | (not estimable) | |  |  | | |  |

dy/dx = average difference in outcome probability between a category and its reference category in a variable

* Four cases who moved from co-living to an institution were excluded because none had weight loss of >10% to compare to the reference (weight loss ≤10%).
